# Deficiency of iPLA_2_β Primes Immune Cells for Proinflammation: Potential Involvement in Age-Related Mesenteric Lymph Node Lymphoma

**DOI:** 10.3390/cancers7040901

**Published:** 2015-12-09

**Authors:** Johannes Inhoffen, Sabine Tuma-Kellner, Beate Straub, Wolfgang Stremmel, Walee Chamulitrat

**Affiliations:** 1 Department of Internal Medicine IV, University Heidelberg Hospital, 69120 Heidelberg, Germany; Johannnes.Inhoffen@med.uni-heidelberg.de (J.I.); Sabine.Tuma@med.uni-heidelberg.de (S.T.-K.); Wolfgang.stremmel@med.uni-heidelberg.de (W.S.); 2 Pathology Institute of Medical Faculty Heidelberg, 69120 Heidelberg, Germany; Beate.Straub@med.uni-heidelberg.de

**Keywords:** Kupffer cells, lymphocytes, immune response, PLA2G6, CD95/FasL, M1 and Th1 cytokines, mesenteric lymph node lymphoma

## Abstract

Proinflammation can predispose the body to autoimmunity and cancer. We have reported that iPLA_2_β^−/−^ mice are susceptible to autoimmune hepatitis and colitis. Here we determined whether cytokine release by immune cells could be affected by iPLA_2_β deficiency alone or combined with CD95/FasL-antibody treatment *in vivo*. We also determined whether cancer risk could be increased in aged mutant mice. Immune cells were isolated from 3-month old male WT and iPLA_2_β^−/−^ mice, and some were injected with anti-CD95/FasL antibody for 6 h. Kupffer cells (KC) or splenocytes and liver lymphocytes were stimulated *in vitro* by lipopolysaccharide or concanavalinA, respectively. Whole-body iPLA_2_β deficiency caused increased apoptosis in liver, spleen, and mesenteric lymph node (MLN). KC from mutant mice showed suppressed release of TNFα and IL-6, while their splenocytes secreted increased levels of IFNγ and IL-17a. Upon CD95/FasL activation, the mutant KC in turn showed exaggerated cytokine release, this was accompanied by an increased release of IFNγ and IL-17a by liver lymphocytes. Aged iPLA_2_β^−/−^ mice did not show follicular MLN lymphoma commonly seen in aged C57/BL6 mice. Thus, iPLA_2_β deficiency renders M1- and Th1/Th17-proinflammation potentially leading to a reduction in age-related MLN lymphoma during aging.

## 1. Introduction

Cancer development has been linked to environmental factors such as tobacco, obesity and infections which affect cancer incidence by 30%, 14%–20%, and 18%, respectively [1]. A common feature connecting all these factors to carcinogenesis is inflammation [1]. Acute inflammation is a necessary requisite in protecting the body against infections, tissue stress and injury. However, gene mutations and genetic background may result in dysregulated metabolism and signaling pathways leading to an inability of the body to return to homeostasis, and also rendering it susceptible to chronic inflammation. It is thought that inflammation changes the homeostatic set points which can promote inflammation further; for an example, hyperglycemia leading to dysmetabolism of glucose toxicity can promote inflammation and tissue damage [2]. Ample evidence has shown that chronic inflammation predisposes the body to chronic diseases and ultimately cancer [1]. The most obvious evidence for this is the predisposition of inflammatory bowel disease and hepatitis B and C infection to cause colon carcinogenesis and hepatocellular carcinoma, respectively [3]. It has been therefore proposed that in order to decrease the cancer risk, the mechanisms of chronic inflammation and predisposition need to be intensively studied, which may provide insights into the development of effective cancer therapies [3].

Genes that play a house-keeping and homeostatic role have been thought to be involved in the predisposition to chronic inflammation. One of these genes is group VIA calcium-independent phospholipase A2 or iPLA_2_β which cleaves a phospholipid at sn-2 position to generate a lysophospholipid and a fatty acid, generally arachidonic acid [4]. Ample data have shown that iPLA_2_β elicits physiologically important functions including phospholipid remodeling, signal transduction, cell proliferation, and apoptosis [5]. An inhibition of this enzyme induces apoptosis [6], partly by preventing arachidonate incorporation into phospholipids [7]. Whole-body deletions of iPLA_2_β (iPLA_2_β^−/−^ mice) have been generated by homologous recombination showing two major phenotyopes with reduced fertility in male mice [8], and defects in insulin secretion by pancreatic β-cells [9]. The latter is caused by the reduction of arachidonate-induced calcium stores in the endoplasmic reticulum, and increased apoptosis in β-cell mitochondria [4]. The lack of the ability of mutant mice to secrete insulin results in the observed sensitization of glucose intolerance upon feeding with high-fat diet or treatment with the β-cell toxin streptozotocin [9]. Upon aging to 1–2 years old, iPLA_2_β^−/−^ mice show a loss of bone mass associated with a decrease in body weight [10], and their brains exhibit neuroaxonal dystrophy [11]. Hence, iPLA_2_β deficiency at the whole body level may elicit systemic effects associated with chronic inflammation in several tissues, and essentially leading to injury such as seen in the bone and brain of aged mutant mice [10,11].

Work in our laboratory has supported the homeostatic role of iPLA_2_β that the livers of iPLA_2_β^−/−^ mice show increased apoptosis associated with increased inflammation [12]. While iPLA_2_β^−/−^ mice physically appear to be normal, they however exhibit *in vivo* susceptibility towards lipopolysaccharide (LPS)-induced liver injury [12], concanavalinA (ConA)-induced autoimmune hepatitis [13], and dextran sodium sulfate induced-colitis [14]. More relevantly, it has been shown that iPLA_2_β mediates apoptotic cell clearance through the generation of lysophosphatidylcholine (LPC) [15], and the enzyme itself has been shown to also regulate the speed and directionality of monocytes during chemotaxis [16]. This defect in apoptotic cell clearance might explain the observed susceptibility of iPLA_2_β^−/−^ mice towards stress-induced injury. It is known that dying cells actively promote their own removal by secreting “find-me” and “eat-me” signals [17]. One such “find-me” signal has been identified as LPC which is produced by activated iPLA_2_β during cleaved caspase 3-mediated apoptosis [15]. A number of studies have also shown that mice deficient in a “find-me” signal exhibit the inability to remove apoptotic cells [18,19]. It is plausible that the lack of LPC during iPLA_2_β deficiency results in an accumulation of apoptotic cells which become secondary necrotic, and subsequently trigger a pro-inflammatory response by immune cells [20,21]. Furthermore, mice lacking G protein coupled receptor 132 (G2A-R) which is thought to be an LPC-receptor have been shown to develop an autoimmune disease with a phenotype similar to systemic lupus erythematodes [22]. Hence, ample data have suggested a homeostatic role of iPLA_2_β likely in immune cells, and that this altered immunity may render the susceptibility for inflammation and injury as observed in our experiments [12,13,14].

It has been recognized that dysregulation of cytokine release during inflammation and infection is an important component in the development of autoimmune diseases and cancer [23,24], particularly those cytokines released by macrophages and T cells [25]. We therefore aimed to determine whether macrophages and lymphocytes isolated from iPLA_2_β^−/−^ mice would exhibit altered cytokine release upon *in vitro* stimulation. As CD95/FasL is capable of inducing proinflammatory cytokines [26], we further studied whether treatment of iPLA_2_β^−/−^ mice with anti-CD95/FasL antibody would cause exaggerated cytokine release by immune cells. Finally, we also determined whether iPLA_2_β deficiency could affect lymphoma incidence of a cancer prone immune organ—mesenteric lymph node (MLN).

## 2. Results and Discussion

### 2.1. Deficiency of iPLA_2_β Increases Apoptosis in Spleen and Primes Splenocytes for Th1/Th17 Response

**Figure 1:**
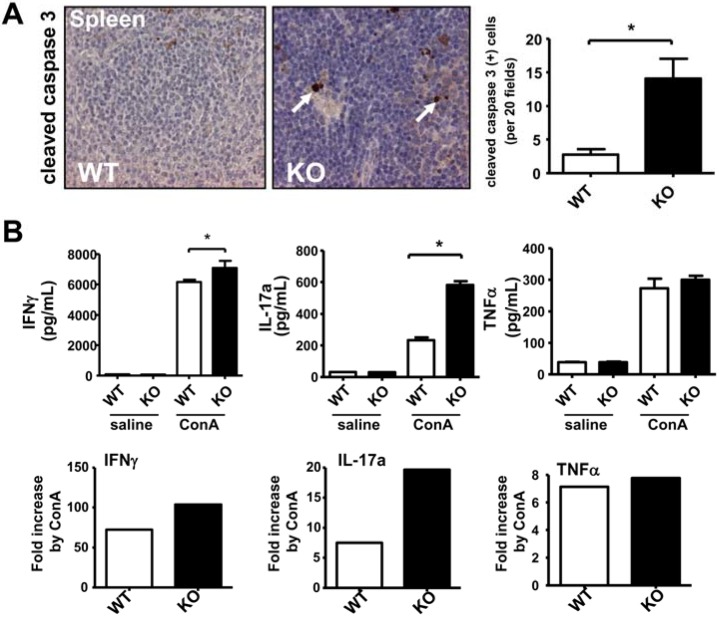
Deficiency of iPLA_2_β increased apoptosis in spleen associated with the sensitized Th1/Th17 release by splenocytes. (**A**) Representative cleaved caspase 3 IHC-staining of a spleen of WT and KO (*left panel*) and its quantification of positive cells (*right panel*). Male mice at 19–24-months old were used (*N* = 8–14 per group); (**B**) ELISA determination of spontaneous and ConA-stimulated release of IFNγ, IL-17, and TNFα (pg/mL) by splenocytes isolated from 3-month old WT and mutant male mice (*N* = 4–6 per group). Saline or 10 µg/mL ConA was used to treat WT and KO splenocytes for 48 h, and a fold-increase of cytokine release by ConA treatment was calculated. * *p* < 0.05 *vs.* WT; ** *p* < 0.005 *vs.* WT.

In terms of inflammation and immune response the spleen holds a unique role in the body. It is the largest secondary lymphoid organ comprising a quarter of the body’s lymphocytes, and the immune responses to blood transmitted antigens are initiated in the spleen [27]. Noteworthily, by immunohistochemical (IHC) staining of cleaved caspase 3, the spleens of aged 19–24 months old male iPLA_2_β^−/−^ (KO) mice displayed a nearly 5-fold increase in the number of apoptotic splenocytes compared with those of control WT mice (Figure 1A). This was accompanied with a 1.6-fold elevation of caspase 3/7 activity in spleen homogenates (Supplementary Figure S1A). As we anticipated that increased apoptosis was a prerequisite for proinflammation in iPLA_2_β-deficient mice [15,16,17,18,19,20,21,22], we determined the functional cytokine release by splenocytes. In young male mice, we observed that iPLA_2_β deficiency did not alter spontaneous cytokine release by splenocytes. However, iPLA_2_β^−/−^ splenocytes exhibited exaggerated release of IFN-γ and IL-17a when stimulated with 10 µg/mL ConA for 48 h (Figure 1B). In particular, ConA treatment stimulated the release of IL-17a by ~8 folds in control splenocytes and by ~20 folds in iPLA_2_β^−/−^ splenocytes, (Figure 1B). ConA stimulation increased the release of TNFα, IL-10, and IL-4 to the same levels among mutant and control splenocytes (Figure 1B, and Supplementary Figure S1B). Thus, our data showed that ablation of iPLA_2_β in young mice primed the splenocytes for Th1/Th17 cytokine release upon ConA stimulation Hence, abnormal Th1/Th17 cytokine release by mutant splenocytes may have rendered autoimmunity [28,29,30], and this was associated with increased apoptosis in spleens of aged mutant mice (Figure 1A).

The observed exaggerated Th1/Th17 cytokine release by mutant splenocytes may also be applied to resident lymphocytes in other organs such as liver and intestine. The activation of these lymphocytes may in turn activate monocytes and macrophages, and further exacerbate tissue injury. This may be the case in our previous report showing increased apoptosis and inflammation associated with liver damage and duodenal enteropathy in ConA-induced autoimmune hepatitis in iPLA_2_β^−/−^ mice [13]. In line with this notion, Crohn’s disease is thought to be caused by Th1/Th17 cell activity [31,32], which might promote cancer development [3]. Furthermore, IL-17a has been shown to be an important promoter of colonic hyperplasia and tumor formation [32] possibly by inducing the release of IL-6 and TNFα [25], and the latter is one of the key proinflammatory cytokines in macrophages [33]. On the contrary, it has been reported that IFN-γ and IL-17a can support growth of fully established cancers by secondarily inducing Th2 lymphocytes or M2 macrophages in repair mechanism [34,35]. On the other hand, IFN-γ may inhibit tumorgenesis as it is shown that IFN-γ double-knockout mice are prone to neoplasms caused by chronic inflammation [35,36]. Thus, we speculate that Th1/Th17 cytokines from mutant splenocytes may promote tumorigenesis during preneoplastic progression, and together with Th2 and M2 cytokines IFNγ may suppress tumorigenesis of full-grown tumors.

### 2.2. iPLA_2_β Deficiency Increases Apoptosis in Liver Associated with Suppressed Cytokine Release by KC

**Figure 2:**
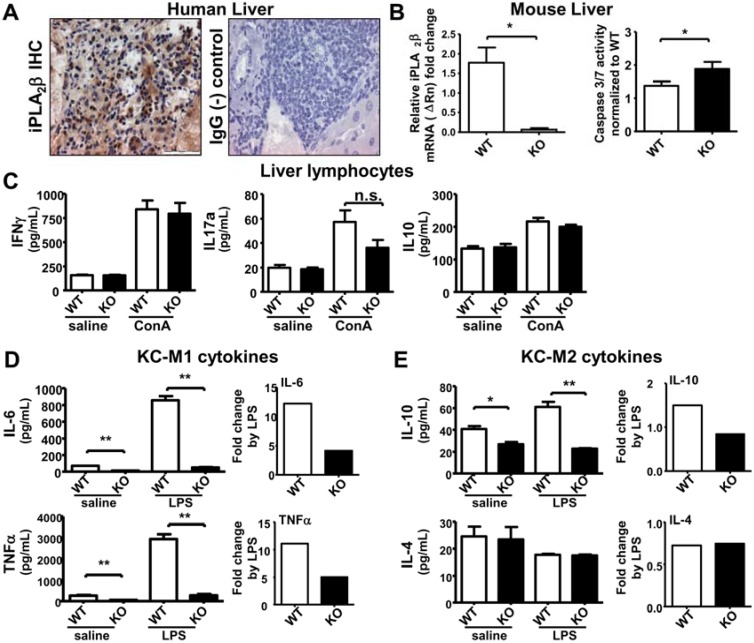
iPLA_2_β deficiency leads to increased hepatic apoptosis associated with suppressed M1 cytokine release by KC either spontaneously or during LPS-stimulation. Male mice at 3 months old were used. Liver lymphocytes were treated with 10 µg/mL ConA for 48 h. KC were treated with 1 µg/mL LPS for 7 h. (**A**) iPLA_2_β IHC of human liver showed positive brown staining. IgG was used as (-) control; (**B**) In left panel, iPLA_2_β mRNA expression in livers of young mice was determined by qRT-PCR. In right panel, caspase 3/7 activity measured by luminescence normalized to the WT levels was obtained in liver homogenates of WT and KO mice (*N* = 3 per group for PCR and *N* = 4–5 per group for luminescence); (**C**) Spontaneous or ConA-stimulated release of IFNγ, IL-17 and IL-10 measured by ELISA was determined in liver lymphocytes of 3-month old WT and KO (*N* = 4–6 per group); Spontaneous or LPS-stimulated release of IL-6 and TNFα (**D**) as well as IL-10 and Il-4 (**E**) measured by ELISA was determined in KC isolated from WT and KO (*left panel*), and their fold increase by LPS was calculated (*right panel*) (*N* = 6 per group). * *p* < 0.05 *vs.* WT; ** *p* < 0.005 *vs.* WT.

Another highly immunologically active organ is the liver which contains the largest group of fixed macrophages in the body which screen antigen-rich blood from the gastrointestinal tract and also phagocytose apoptotic hepatocytes [37,38]. From iPLA_2_β IHC staining of human livers, we could demonstrate that iPLA_2_β was expressed ubiquitously in hepatocytes and immune cells (Figure 2A). Regrettably, our antibodies against iPLA_2_β did not work with mouse liver tissues. By quantitative RT-PCR, however, we could not detect any iPLA_2_β mRNA in livers of iPLA_2_β-deficient mice suggesting a depletion of iPLA_2_β in immune cells (Figure 2B, left panel). Similar to the observation in spleen, a significant increase of apoptosis (caspase 3/7 activity) was observed in liver homogenates of 3-month old iPLA_2_β^−/−^ mice (Figure 2B, right panel), which is consistent with our previous study in 12-month old mutant mice [12].

We further determined whether iPLA_2_β deficiency could affect cytokine releases by liver lymphocytes and Kupffer cells (KC). Liver lymphocytes isolated from iPLA_2_β^−/−^ mice did not alter the release of IFN-γ, IL-17a, and IL-10 either spontaneously or upon ConA stimulation when compared with those from control mice (Figure 2C). Unlike splenocytes iPLA_2_β deficiency alone did not have any effects on liver lymphocytes regarding cytokine release.

Macrophages are categorized into pro-inflammatory M1 or anti-inflammatory M2 macrophages [39]. We here presented our data on KC cytokine release by categorizing into M1-related and M2-related cytokines. Intriguingly, KC isolated from iPLA_2_β^−/−^ mice spontaneously secreted M1- and M2-related cytokines at lower levels than those from control mice (Figure 2D,E). To better visualize the effect, we stimulated KC *in vitro* with 1 µg/mL LPS for 7 h. Upon LPS stimulation, the mutant KC released IL-6 and TNFα at significant lower levels than control KC (Figure 2D). This suppressed release was also observed in the release of IL-10 (but not IL-4) by mutant KC (Figure 2E). It is postulated that apoptotic hepatocytes (Figure 2B) may render suppressive effects on mutant KC to release M1 and M2 cytokines (Figure 2D,E). A previous study has shown that macrophages treated with LPS in the presence of apoptotic cells showed suppressed pro- and anti-inflammatory cytokine release after 24 h [40]. This time delay may be required to serve the purpose of limiting inflammatory response in damaged tissue thereby preventing chronic inflammation and an initiation of resolution [41,42]. Especially important in this context is the decreased release of TNFα by KC of young mutant mice. Increased TNFα contributing to chronic inflammation is observed in livers of older 12-month old mutant mice [12], and in this case, cancer development may be amplified at older age [25,33].

Alternatively, the inability of mutant KC to respond to LPS for the release of M1 and M2 cytokines may also indicate a defect in innate immunity due to iPLA_2_β deficiency. This may lead to the inability of mutant mice to respond to infection, again rendering sensitization for further injury. In line with this, it has been shown that peritoneal macrophages isolated from iPLA_2_β^−/−^ mice are unable to respond to viral infection associated with suppressed expression of inducible nitric oxide synthase [43]. Thus, the defect of mutant KC to secrete M1 cytokines may reflect immunosuppression triggered by iPLA_2_β deficiency.

### 2.3. Sublethal Dose CD95/FasL Treatment Primes Mutant KC for Enhanced M1 Cytokine Release

**Figure 3:**
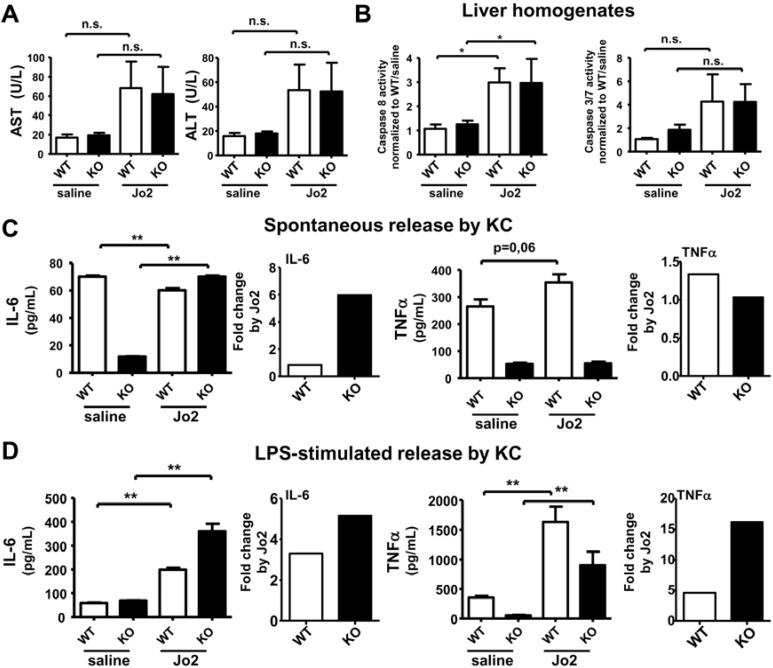
Sublethal dose Jo2 treatment caused a very mild effect on liver injury and apoptosis but primes KC from mutant mice for a marked increase of IL-6 release either spontaneously or during LPS stimulation. Three-month old mice were treated with saline or 0.125 µg/g body weight Jo2 antibody for 6 h. KC were treated with saline or 1 µg/mL LPS for 7 h. (**A**) Activity of serum transaminases (AST and ALT) in U/L were determined in WT and KO mice (*N* = 3–7 per group); (**B**) Caspase 8 and caspase 3/7 activities measured by luminescence were determined in liver homogenates of WT and KO (*N* = 3–7 per group); Spontaneous (**C**) and LPS-stimulated (**D**) release of IL-6 and TNFα measured by ELISA was determined in KC isolated from WT and KO (*N* = 6 per group), and the fold increase by Jo2 was calculated (right-hand panel). * *p* < 0.05 *vs.* untreated; ** *p* < 0.005 *vs.* untreated.

It is known that the ligation of CD95/FasL receptor (by Jo2 antibody for mouse and CH11 antibody for humans) on circulating monocytes and tissue macrophages can induce pro-inflammatory cytokine response that initiates tissue injury [26,44]. Interestingly, this pro-inflammatory effect by CD95/FasL is caspase dependent, but apoptosis independent [26]. As we found that mutant-derived KC released decreased levels of cytokines (Figure 2), we therefore further determined whether Jo2 treatment *in vivo* could prime KC isolated from iPLA_2_β^−/−^ mice for proinflammatory response *in vitro*. A high dose of 0.25 µg/g body weight Jo2 antibody normally kills mice within four hours [45]. In our experiments, we used a sublethal dose at 0.125 µg/g body weight Jo2 antibody, and mice were sacrificed 6 hours later. This low dose treatment failed to significantly induce any increases in serum transaminases (AST and ALT) in control and iPLA_2_β^−/−^ mice (Figure 3A). The extent of apoptosis measured by caspase 3 activity in liver homogenates of control and mutant mice was also not significantly affected (Figure 3B, right panel). However, Jo2 treatment caused a marked increase in caspase 8 activity by 3 folds in mutant and control mice alike (Figure 3B, right panel).

Transaminase elevation is normally observed with Jo2 administration reflecting hepatotoxicity, however this elevation has been shown to be independent from apoptosis in mouse liver [46,47]. In another study, low dose Jo2 administration does not necessarily elevate ALT levels [48]. Our low-dose Jo-2 treatment elicited only a moderate effect in elevating hepatic caspase 8 activity, but did not sufficiently elevate serum transaminases, and hepatic caspase 3 activity. However this mild effect of Jo2 apparently could prime immune cells for cytokine release.

For spontaneous cytokine release by KC, we observed that Jo2 administration led to a strong (6-fold) increase of IL-6 release by mutant KC, whereas it had a suppressive effect on WT KC (Figure 3C). Mutant KC responded to Jo2 for TNFα secretion to a slight lesser extent than control KC. This weak effect by Jo2 treatment was consistent with a previous report that spontaneous release of TNFα by macrophages was not affected by CD95/FasL treatment [26]. We found that the response to Jo2 for spontaneous IL-10 and IL-4 release was the same between mutant and control KC (Supplementary Figure S2A), indicating a lack of effects on M2-cytokines by *in vivo* Jo2.

For LPS-stimulated release, both mutant and control KC responded to Jo2 treatment with a marked increase of both IL-6 and TNFα, while the mutant KC responded to Jo2 with a much greater extent than control KC as seen by fold change by Jo2 (Figure 3D). For LPS-stimulated release, we found that the response to Jo2 for IL-10 and IL-4 release was the same between mutant and control KC (Supplementary Figure S2B), again indicating no role of Jo2 on M2 release.

Collectively, Jo2 administration at nonlethal dose was able to prime iPLA_2_β-deficient KC for an exaggerated increase in M1 cytokines in an absence of overt liver injury and apoptosis (Figure 3A,B). The exaggerated stimulated release of TNFα and IL-6 by mutant KC is in line with our previous findings that the duodenal enteropathy in ConA-treated iPLA_2_β^−/−^ mice is mainly due to TNFα and IL-6 from macrophage activation detected in the intestine [13].

### 2.4. Sublethal-Dose CD95/FasL Treatment Primes Mutant Liver Lymphocytes for a Weak Increase in Th1 Cytokine Release

In addition to KC, Jo2 administration *in vivo* may prime other immune cells for cytokine release as well [49]. Thus, we further determined the cytokine release by liver lymphocytes and splenocytes in control and iPLA_2_β^−/−^ mice treated with Jo2.

**Figure 4:**
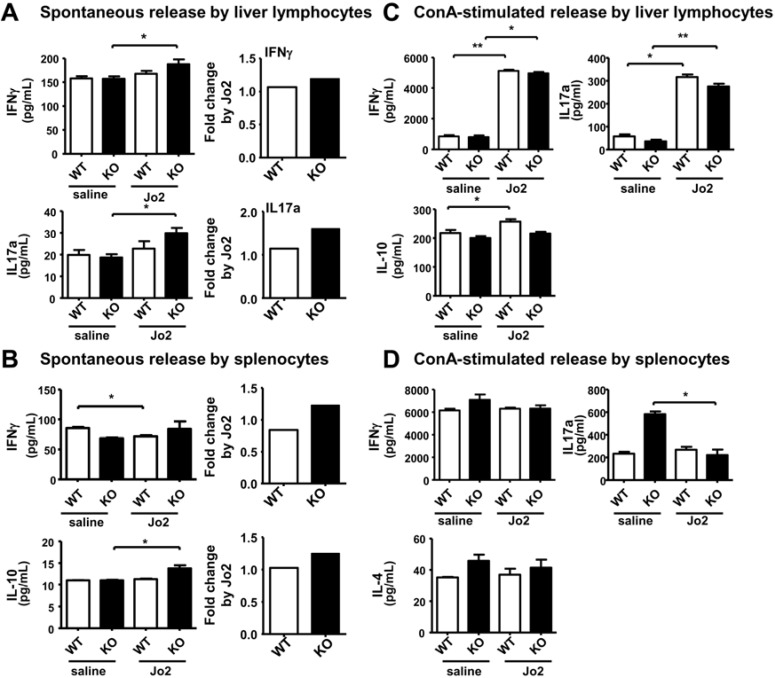
Sublethal *in vivo* Jo2 treatment of KO mice led to a weak increase in Th1 cytokine release by liver lymphocytes and splenocytes. Three-month old mice were treated with saline or 0.125 µg/g body weight Jo2 antibody for 6 h. (**A**) The spontaneous release of IFNγ and IL-17a measured by ELISA was determined in liver lymphocytes isolated from WT and KO (*N* = 4–5 per group); (**B**) The release of IFNγ and IL-10 measured by ELISA was determined in splenocytes of WT and KO (*N* = 5–6 per group); (**C**) The ConA-stimulated release of IFNγ, IL-17aγ, and IL-10 was determined in liver lymphocytes from WT and KO (*N* = 6 per group); (**D**) The ConA-stimulated release of IFNγ, IL-17a, and IL-4 was determined in splenocytes of WT and KO (*N* = 4 per group). * *p* < 0.05 *vs.* control; ** *p* < 0.005 *vs.* control.

For spontaneous release, liver lymphocytes isolated from iPLA_2_β^−/−^ mice responded to Jo2 treatment by moderately increasing the release IFN-γ and IL-17a by ~1.3–1.5 fold when compared with those cells isolated from control mice (Figure 4A). Splenocytes from iPLA_2_β^−/−^ mice responded similarly to Jo2 with a ~1.3-fold increase of IFN-γ and IL-10 release (Figure 4B). For spontaneous release, we could observe that a weak extent of Jo2 response for Th1 cytokine release by mutant liver lymphocytes (Figure 4A,B), while a much stronger increase was observed for M1 cytokine release by mutant KC (Figure 3C).

For stimulated release by ConA *in vitro*, liver lymphocytes isolated from Jo2-treated control and iPLA_2_β^−/−^ mice secreted IFN-γ and IL-17a to the same extent (Figure 4C). Similarly, ConA-treated splenocytes isolated from Jo2-treated control and mutant mice released IFN-γ and IL-4 to the same extent (Figure 4D); whereas there was a suppressive release of IL-17a by mutant splenocytes upon Jo2 treatment. For stimulated release, there was no difference on Jo2 response among mutant and control liver lymphocytes (Figure 4C,D), whereas a much stronger increase was observed for M1 cytokine release by mutant KC (Figure 3D).

As autoimmune hepatitis is associated with an increased release of IFN-γ and IL-17a [50,51], the weak increase of spontaneous and stimulated release of Th1-cytokines by liver lymphocytes from iPLA_2_β^−/−^ mice is consistent with our reported susceptibility of iPLA_2_β^−/−^ mice to ConA-induced autoimmune hepatitis [13]. Regarding Jo2 treatment *in vivo*, macrophages and KC appeared to be the first line of cells which were strongly sensitized by iPLA_2_β deficiency, and these primed cells may have in turn elicited sensitized effects onto liver lymphocytes with a lesser extent in response pattern.

### 2.5. Mesenteric Lymph Node Abnormalities of Aged iPLA_2_β-Deficient Mice

As iPLA_2_β^−/−^ mice progressively develop abnormalities in the bone, and brain at 1–2 years of age, we were interested in investigating whether there were any abnormalities in the mesenteric lymph nodes (MLN) of mutant mice at old age, since this organ is prone for neoplastic development [52]. We allowed our mouse colonies to age to 19–24 months old during 2012–2014. Unfortunately, we later discovered that the rooms where our transgenic mice were housed during this time were infected with *Helicobacter hepaticus* (*H. hepaticus*). This could have an impact on our results and their interpretation as discussed below.

**Figure 5:**
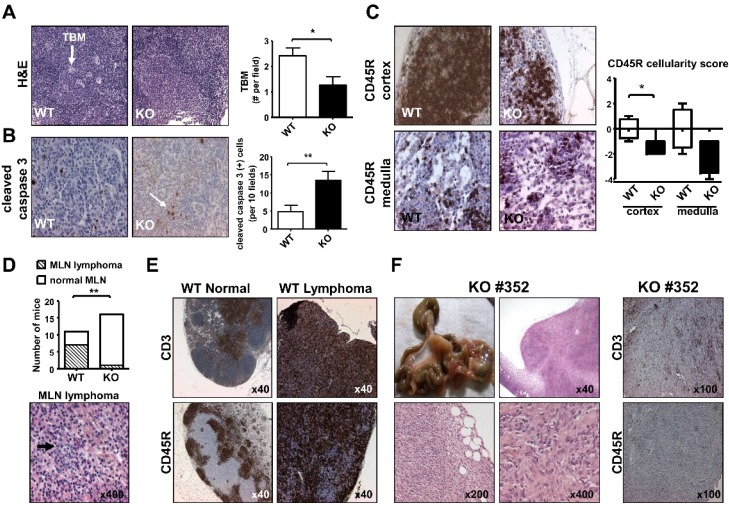
iPLA_2_β deficiency caused MLN apoptosis, loss of B cell cellularity, reduction in the incidence of follicular center cell lymphoma, and a rare MLN histiocytosis. Male mice at 19–24 months old were used (**A**) Representative H&E staining showed tingible body macrophages (TBM indicated by a white arrow) in the germinal centers of WT and KO (*left panel*), and TBM quantification (*right panel*) (*N* = 6–8 per group); (**B**) Representative cleaved caspase 3 IHC (indicated by a white arrow) showed apoptosis in WT and KO MLN (*left panel*), and apoptotic cell quantification (*right panel*) (*N* = 9 per group); (**C**) Representative CD45R IHC staining showed cellularity of cortex and medulla of WT and KO MLN (*left panel*), and CD45R (+) quantification (*right panel*) (*N* = 3–4 per group for medulla, and *N* = 5–9 per group for cortex); (**D**) Quantification of lymphoma incidence (*N* = 11–16 per group, upper panel). Bottom panel shows H&E of MLN follicular lymphoma from a WT mouse with centroblasts indicated by an arrow; (**E**) Representative CD3 (top) and CD45R (bottom) IHC staining of from left to right normal and lymphoma of MLN of WT mice. (F) Macroscopic picture, H&E and MLN lymphoma of KO#352 mouse. * *p* < 0.05 *vs.* control; ** *p* < 0.005 *vs.* control; ×40, ×100, ×200, ×400 magnification.

We screened 16 mutant and 11 control aged male mice for monitoring possible MLN neoplasm. The first result on H&E staining of MLN showed that the number of tingible body macrophages (TBM) located in the germinal center was decreased in iPLA_2_β^−/−^ mice, when compared with that in control mice (Figure 5A). This decrease would result in an impaired engulfment of apoptotic cells by TBM, and this was consistently observed by an increase of IHC staining of cleaved caspase 3 in mutant MLN (Figure 5B). Concomitantly, there was a severe reduction in cellularity score with the CD45R IHC staining in the cortex and medulla of iPLA_2_β^−/−^ MLN (Figure 5C), while CD3 IHC staining was unaffected by iPLA_2_β deficiency (Supplementary Figure S3). The loss of cellularity of CD45R or B (+) cells in mutant MLN may indicate an abnormality in adaptive immunity. This is in line with the inability of mutant KC to secrete M1 and M2 cytokines indicating abnormal innate immunity (Figure 2C,D).

We inherently observed that aged WT male mice showed strikingly high rates of follicular center cell lymphoma of MLN (Figure 5D, top panel). This lymphoma is the most common malignancy in C57/BL6 mice known to be of B cell origin [52]. In the light of *H. hepaticus* infection in our animal facility, we can only speculate about the impact of this on our data. C57/BL6 mice are typically resistant to damage caused by *H. hepaticus* [53]. *H. hepaticus* infection in mice causes an increase of IFN-γ and IL-17a levels and shown to be linked to hepatitis, colitis and colon cancer [54]. Thus, the high incidence of MLN follicular center cell lymphoma in aged male WT mice may not have been due to *H. hepaticus* infection.

The WT MLN follicular lymphoma was typically composed of centroblasts and centrocytes at various proportions with various sizes and shape (Figure 5D, bottom panel with centroblasts indicated by an arrow). Compared with normal MLN from WT mice, this lymphoma exhibited the loss of MLN architecture as indicated by CD3 and CD45R staining (Figure 5E). The CD45 IHC staining of follicular lymphoma in WT MLN was more strongly and uniformly than that of normal WT MLN (Figure 5E), supporting the active proliferation of B cells in follicular lymphoma.

The incidence of MLN follicular lymphoma was found to be decreased in aged iPLA_2_β^−/−^ male mice (Figure 5D, top panel).The decreased incidence of follicular center cell lymphoma in aged mutant mice was consistent with decreased B cell cellularity in iPLA_2_β^−/−^ MLN (Figure 5C). This decreased incidence could be further explained by the homeostatic role of iPLA_2_β in lymphocyte proliferation [55], whereby the full-grown lymphoma cells have adapted themselves to utilize iPLA_2_β for their rapid hyperplastic growth [56].

Furthermore, it has been shown that IFN-γ is an important factor in suppressing lymphoma development [57], but on the other hand, IL-6 and TNFα are tumor promoting [35]. IL-6 especially plays a pivotal role in *in vivo* B cell neoplasm genesis [58]. The alteration in cytokine release by immune cells from young mutant mice showing an increase of IFN-γ (by mutant splenocytes), and decreased IL-6 and TNFα (by mutant KC) may provide a microenvironment that does not support the development of MLN lymphoma in aged iPLA_2_β^−/−^ mutant mice.

Notwithstanding this, among 16 aged iPLA_2_β^−/−^ male mice, only one mutant MLN (KO#352) showed a small hyperplasia being different from follicular center cell lymphoma (Figure 5F). This hyperplasia showed a loss of MLN architecture with no discernible structure of cortex, paracortex, subcapsular sinus as well as medullary cords and sinuses. At higher magnification of KO#352 MLN, a sheet of elongated or spindle cells with fusiform in appearance could be observed, and these lymphoma cells were multinucleated (Figure 5F, bottom panel). These cells could be of non-lymphoid and myeloid origin [59]. We speculate that KO#352 MLN hyperplasia could be histiocyte-associated large B cell lymphoma [52] or histiocytic sarcoma [59]. However, the lymphoma seen in KO#352 MLN was neither CD3- nor CD45R-positive (Figure 5F), suggesting that these lymphoma cells were not of T nor B cell origin. The incidence of histiocytic sarcoma is occasionally seen in MLN of C57/BL6 mice [52]. We however did not observe any abnormalities in 11 MLN of aged control male mice.

We also observed abnormalities in the liver of KO#352 mice showing an enlargement of hepatocytes, the presence of inclusions body in hepatocyte nuclei, and marked infiltration of immune cells (data not shown). This indicates that KO#352 had a systemic inflammation possibly by histiocytes invading into the liver. It is known that *H. hepaticus* infected mice develop lympho-histiocytic infiltrates in the hepatic parenchyma [60], and CYP1B1-deficient mice that are inefficient at removing apoptotic and necrotic cells can develop high incidence of histiocytic sarcoma during *H. hepaticus* infection [61]. Therefore, deficiency of iPLA_2_β in this particular KO#352 may have abrogated the known protective nature of C57/BL6 mice against *H. hepaticus* [53]. We thus speculate that the MLN histiocytic sarcoma seen in KO#352 may have been due to *H. hepaticus* sensitization. Further experiments are warranted to confirm whether deliberately infecting iPLA_2_β-deficient mice with *H. hepaticus* would lead to the development of MLN histiocytic hyperplasia.

Taken together, with aging an increase in apoptosis in MLN was associated with an inherent loss of B cell cellularity, decreased amount of lymphoma and in one exceptional mutant mouse, *i.e.*, KO#352, a rare histiocytic lymphoma could be formed.

## 3. Experimental Section

### 3.1. Animals and Treatment

We received iPLA_2_β-null (iPLA_2_β^−/−^) mice as a kind gift from Dr. John Turk (Washington University School of Medicine, St. Louis, MO, USA). Mice were bred with C57/B6L background, and genotyped based on published work [9]. All mice were kept at the animal facility of the University of Heidelberg. iPLA_2_β^+/+^ WT mice were used as control. Two different sets of experiments were performed. To assess the influence of aging, 19–24 months old male mice were used. For measurement of cytokine release by isolated immune cells, male mice at 3–7 months old were used. Apoptosis was induced by an intraperitoneal injection of CD95/FasL antibody (purified Hamster Anti-mouse CD95 obtained from BD Bioscience, Heidelberg, Germany) at a concentration of 0.125 µg/g body weight solute in saline containing 0.1% BSA. Six hours later mice were killed and blood, liver, spleen, MLN, kidney, lung and thymus were harvested. All experiments were approved by the Animal Care and Use Committee of the University of Heidelberg.

### 3.2. Biochemical Assays

To avoid hemolysis, plasma samples were centrifuged at 1500 rpm for 10 min at 4 °C and the supernatants were collected and stored at −20 °C or 4 °C until use [62,63]. The activities of aspartate aminotransferase (AST) and alanine aminotransferase (ALT) were measured using diagnostic kits from Randox (Krefeld, Germany). For determination of apoptosis activity in tissue homogenates, caspase3/7^Gl0^ and caspase8^Gl0^ kits (Promega, Mannheim, Germany) was used by measuring luminescence over 200 sec with a Lumat LB 9507 (Berthold Technologies, Bad Wildbad, Germany).

### 3.3. Histology and Immunohistochemistry (IHC)

Spleen and MLN were fixed overnight using Bouin’s solution (Sigma, Taufkirchen, Germany). The fixed tissue specimens were embedded in paraffin blocks and cut into 3-µm sections. The sections were stained with hematoxylin and eosin (H&E) for histology. For IHC, following deparaffinizing and re-hydration, specimens were subjected to heat-induced epitope retrieval by heating to 95–98 °C for 20 min in 10 mM citrate buffer (pH 6.0). Samples were treated with 3% H_2_O_2_ and blocked with PBS containing 10% serum for 1 h at room temperature. Sections were incubated with a primary antibody overnight. Cleaved caspase 3 antibody (dilution 1:300) was obtained from Cell signaling (Frankfurt, Germany), and CD45R (1:100) and CD3 (1:200) antibody were obtained from Abcam (Cambridge, UK). For secondary antibody, an avidin-biotin complex kit from Abcam was used to stain cleaved caspase 3, whereas a goat anti-rat (Santa Cruz Biotechnology, Heidelberg, Germany) and a goat anti-rabbit (Abcam) secondary antibody was used to stain CD45R and CD3, respectively. Staining was detected using diaminobenzidine and slides were counterstained with hematoxylin prior to mounting. Slides were evaluated by an Olympus microscope.

Human liver slides were provided by the tissue bank of the National Center for Tumor Disease (NCT, Heidelberg, Germany) in accordance with the regulations of the tissue bank and the approval of the ethics committee of the University of Heidelberg. IHC staining was performed with an antibody against iPLA_2_β (1:250) (Santa Cruz Biotechnology). Mouse IgG (1:1000) was used as a negative control (Santa Cruz Biotechnology).

### 3.4. Cell Isolation and Cell Culture

KC were isolated accordingly to published procedure [64]. Briefly after anesthesia, mouse liver was perfused to remove blood followed by perfusion with buffer containing collagenase. The liver was passed through a 100-µm cell strainer (BD Bioscience). After centrifugation at 20× *g* for 2 min, supernatants were collected and subjected the lysis of red blood cells using hypotonic sodium chloride. KC in supernatants were purified by gradient separation by using Optiprep (Axis-shield, Dundee, Scotland) at 24%, 17%, 11.5% and 8.4% iodixanol. Following 20-min centrifugation at 1400× *g*, KC were retrieved from the interphase between 24%–17% and 17%–11.5%. KC were resuspended in DMEM containing 10% FCS at 1 × 10^6^ cells/mL, and plated in a 96-well plate. After 24-h incubation in a 5% CO_2_/37 °C incubator, KC were treated with E. coli LPS (Sigma) at 1 µg/mL for 7 h. The medium was collected and stored at 20 °C until use.

Splenocytes were isolated as previously described [65]. Briefly, spleens were removed and passed through a 70-µm cell strainer. Red blood cells were removed by using Tris-NH_4_Cl. Cells were resuspended in RPMI-1640 containing 10% FCS at 2.5 × 10^6^ cells/mL, and plated in a 24-well plate. Cells were treated with or without 10 µg/mL ConA (Sigma) for 48 h in a 5%-CO_2_ incubator at 37 °C. The medium was collected and stored at 20 °C until use.

Liver lymphocytes were isolated as previously described [66]. Briefly, the liver was perfused as described earlier, and was passed through a 70-µm cell strainer. Detached cells from the liver were resuspended in 35% Percoll (GE Healthcare Bio-sciences, Uppsala, Sweden) containing 100 U/mL heparin, and subsequently centrifuged at 500× *g* for 15 min. Red blood cells were removed by using Tris-NH_4_Cl. Cells were resuspended in complete RPMI medium and plated at 2.5 × 10^6^ cells/mL in a 24-well plate. Further treatment was the same as for splenocytes.

### 3.5. Determination of Cytokine Release

The levels of IL-10, IL-6, TNFα, IL-17a, and IFNγ in medium were determined by using ELISA kits obtained from BioLegend (Cologne, Germany). ELISA kits for determination of IL-4, IL-6, and TNFα were obtained from eBioscience (Frankfurt, Germany). Supernatants of cultured immune cells with or without stimulation were measured, and a standard curve was run to normalize to pg/mL. All experiments were performed in two independent experiments to establish reproducibility.

### 3.6. Quantitative qRT-PCR

Total RNA was isolated from liver by using Gen Elute™ Miniprep Kit from Sigma (Steinheim, Germany). Five micrograms RNA were converted to cDNA by using a cDNA synthesis kit from Thermo Scientific (Karlsruhe, Germany). Gene expression was analyzed by quantitative real-time polymerase chain reaction (qRT-PCR) using Applied Biosystems TaqMan^®^ gene expression assays with Roche Probes Master mix, and run on a Roche 480. Expression level of target gene in triplets was calculated using Δ–Ct transformation method, and normalized to house-keeping gene GAPDH.

### 3.7. Statistics

All results were plotted as mean ± SEM, and *p* < 0.05 was considered significant. Significance was determined with Mann-Whitney Test using GraphPad Prism 5.

## 4. Conclusions

In conclusion, the major effect upon iPLA_2_β deletion was an induction of apoptosis in immunologically active organs, namely, liver, spleen, and MLN. This led to multiple abnormalities including immunosuppression of M1-releated KC cytokine release, and the priming of splenocytes to release pro-inflammatory Th1/Th17-related cytokines. Mutant KC and to a lesser extent liver lymphocytes showed pro-inflammatory M1 phenotype upon Jo2 stimulation *in vivo*. These data are consistent with our previous findings showing susceptibility of mutant mice to chronic inflammatory diseases [13,14]. Upon aging, MLN of mutant mice showed loss of B cell cellularity with a lesser incidence of follicular lymphoma than control mice. However, a rare histiocytic lymphoma was found in a mutant mouse. Our work may highlight a possibility to screen for iPLA_2_β mutation in human chronic inflammatory diseases, and correlate with disease progression and clinical activity. Because an impairment of apoptotic cell clearance is the main feature of iPLA_2_β deficiency and chronic inflammatory diseases can predispose to cancer genesis, it may be pertinent to investigate along this line more extensively in regards to tumor risk [67]. We are now testing whether iPLA_2_β^−/−^ mice would be susceptible for nitrosamine-induced liver cancer in a chronic model.

## Supplementary Materials

Supplementary figures are available online at http://www.mdpi.com/2072-6694/7/4/0901/s1.
