# Supplementary material for: Deficiency of iPLA2β Primes Immune Cells for Proinflammation: Potential Involvement in Age-Related Mesenteric Lymph Node Lymphoma
Source: Cancers (Basel). 2015 Dec 9;7(4):2427–42. doi: 10.3390/cancers7040901 (PMC4695901; doi:10.3390/cancers7040901)
Supplement: Supplementary file 1 [file cancers-07-00901-s001.pdf]

Supplementary Materials: Deficiency of iPLA₂β Primes Immune Cells for Proinflammation: Potential Involvement in Age-Related Mesenteric Lymph Node Lymphoma

Johannes Inhoffen, Sabine Tuma-Kellner, Beate Straub, Wolfgang Stremmel amd Walee Chamulitrat

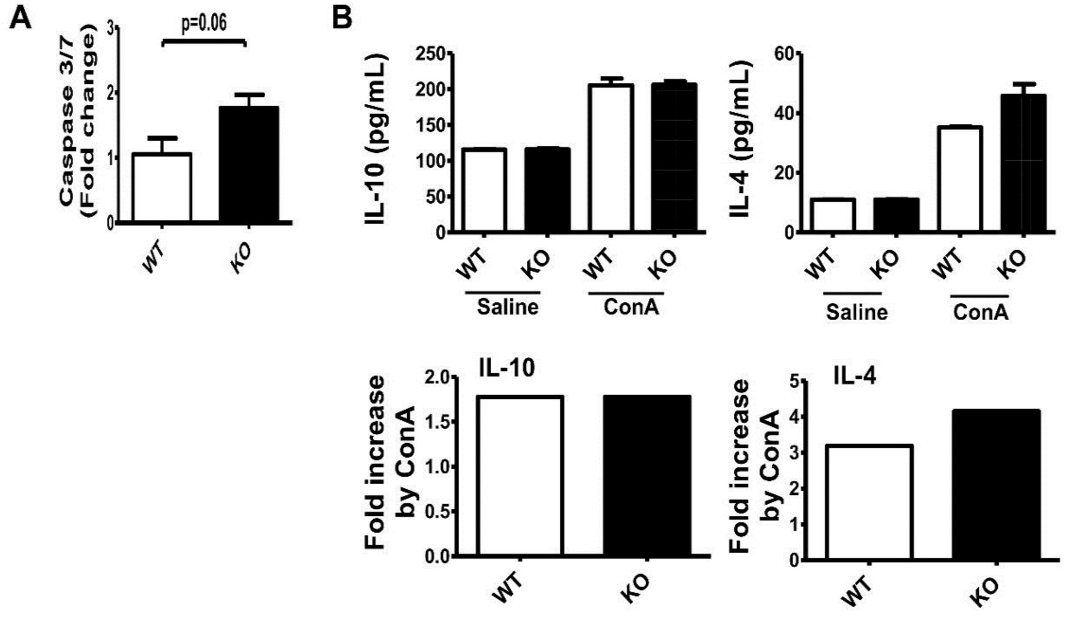

Figure S1. Deficiency of iPLA$_2$β increases caspase 3/7 activity in spleen, but does not significantly prime splenocytes for IL-10 and IL-4 release.

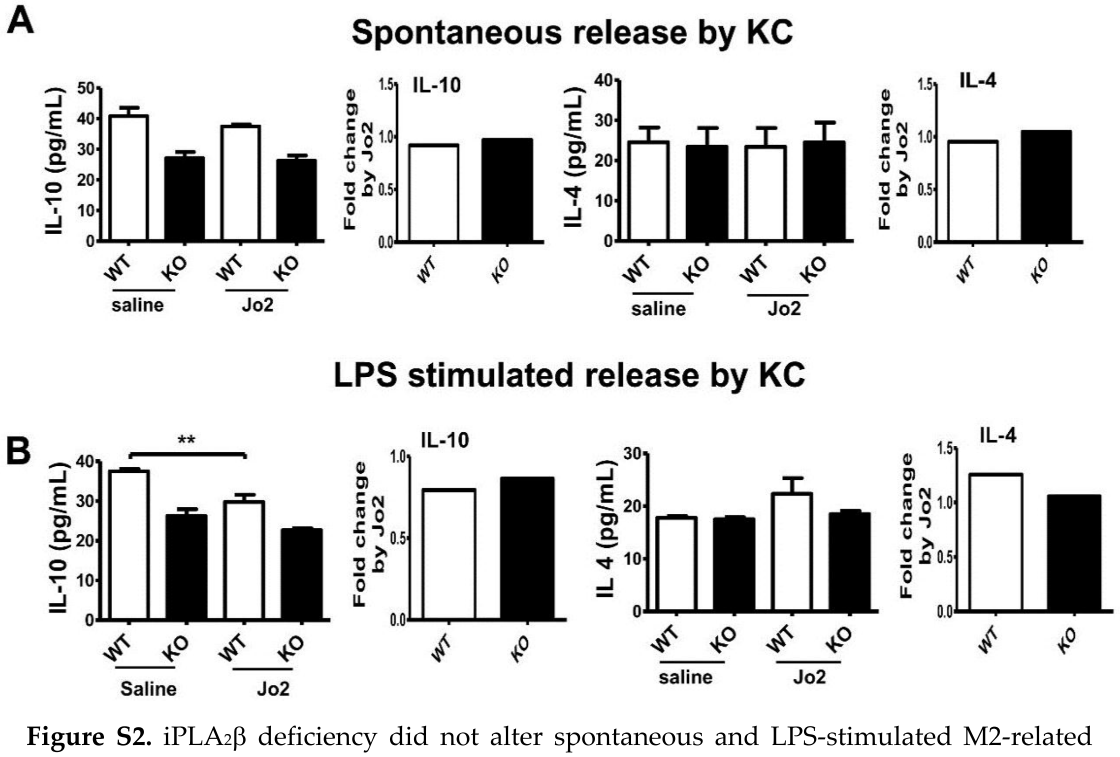

Figure S2. iPLA$_2$β deficiency did not alter spontaneous and LPS-stimulated M2-related cytokine release.

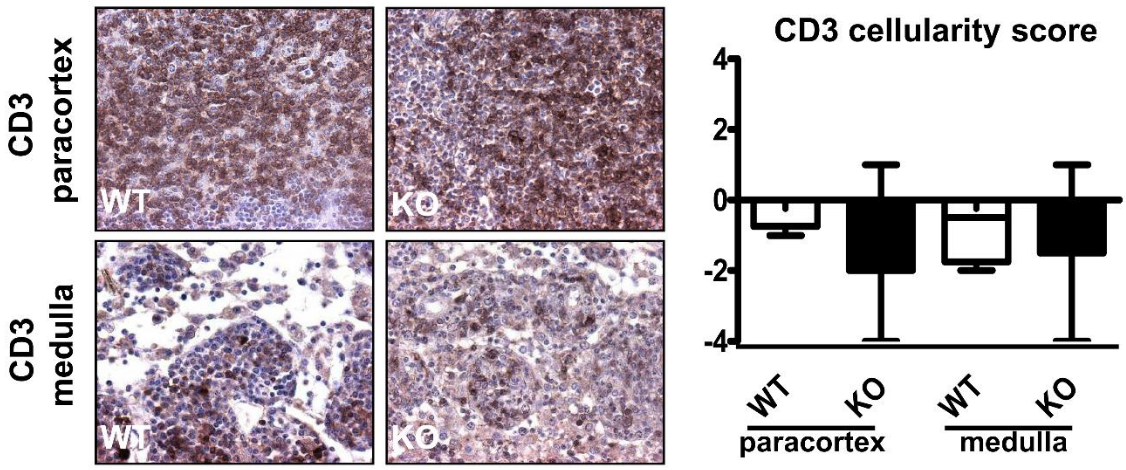

Figure S3. CD3 cellularity in MLN was unaffected by knockout of iPLA$_2$β.
